# The Smart Aerial Release Machine, a Universal System for Applying the Sterile Insect Technique

**DOI:** 10.1371/journal.pone.0103077

**Published:** 2014-07-18

**Authors:** Ruben Leal Mubarqui, Rene Cano Perez, Roberto Angulo Kladt, Jose Luis Zavala Lopez, Andrew Parker, Momar Talla Seck, Baba Sall, Jérémy Bouyer

**Affiliations:** 1 Servicios aéreos Biologicos y Forestales Mubarqui, Cuidad Victoria, Mexico; 2 Programa Moscamed, Tapachula, Mexico; 3 Insect Pest Control Laboratory, Joint Food and Agriculture Organization/International Atomic Energy Agency (FAO/IAEA) Programme of Nuclear Techniques in Food and Agriculture, Vienna, Austria; 4 Institut Sénégalais de Recherches Agricoles, Laboratoire National d’Elevage et de Recherches Vétérinaires, Dakar – Hann, Sénégal; 5 Direction des Services Vétérinaires, Dakar, Sénégal; 6 Unité Mixte de Recherche Contrôle des Maladies Animales Exotiques et Emergentes, Centre de Coopération Internationale en Recherche Agronomique pour le Développement (CIRAD), Montpellier, France; 7 Unité Mixte de Recherche 1309 Contrôle des Maladies Animales Exotiques et Emergentes, Institut national de la recherche agronomique (INRA), Montpellier, France; Natural Resources Canada, Canada

## Abstract

**Background:**

Beyond insecticides, alternative methods to control insect pests for agriculture and vectors of diseases are needed. Management strategies involving the mass-release of living control agents have been developed, including genetic control with sterile insects and biological control with parasitoids, for which aerial release of insects is often required. Aerial release in genetic control programmes often involves the use of chilled sterile insects, which can improve dispersal, survival and competitiveness of sterile males. Currently available means of aerially releasing chilled fruit flies are however insufficiently precise to ensure homogeneous distribution at low release rates and no device is available for tsetse.

**Methodology/Principal Findings:**

Here we present the smart aerial release machine, a new design by the Mubarqui Company, based on the use of vibrating conveyors. The machine is controlled through Bluetooth by a tablet with Android Operating System including a completely automatic guidance and navigation system (MaxNav software). The tablet is also connected to an online relational database facilitating the preparation of flight schedules and automatic storage of flight reports. The new machine was compared with a conveyor release machine in Mexico using two fruit flies species (*Anastrepha ludens* and *Ceratitis capitata*) and we obtained better dispersal homogeneity (% of positive traps, p<0.001) for both species and better recapture rates for *Anastrepha ludens* (p<0.001), especially at low release densities (<1500 per ha). We also demonstrated that the machine can replace paper boxes for aerial release of tsetse in Senegal.

**Conclusions/Significance:**

This technology limits damages to insects and allows a large range of release rates from 10 flies/km^2^ for tsetse flies up to 600 000 flies/km^2^ for fruit flies. The potential of this machine to release other species like mosquitoes is discussed. Plans and operating of the machine are provided to allow its use worldwide.

## Introduction

Area-wide integrated pest management (AW-IPM) has become a prevalent paradigm for insect pest control over the past 50 years [1]. It relies on a good understanding of the ecology of the target populations and optimal integration of both chemical and biological control measures. Only through such an integrated and holistic approach will greater control efficacy and concurrent reduced use of insecticides be achieved. The control of vector-borne diseases in particular is becoming increasingly problematic due to invasions or resurgence of vectors and the necessity to reduce the use of insecticides in view of their environmental and health impacts [2]. Avoiding the negative impacts of reduced insecticide use across large territories necessarily requires an integrative, process-based, preventive and area-wide approach to pest and vector control. Integrated pest management is based on the understanding that each control technique has its advantages and drawbacks (in particular, some techniques are efficient at high densities of the target population whereas others possess an inverse density-dependence efficacy) [3] and that appropriate combination of these techniques (biological, cultural, genetic and chemical) depend on context, taking into consideration the predicted economic, ecological and sociological consequences, with special attention paid to environmental concerns [1].

The sterile insect technique (SIT) and the use of parasitoids are presently among the biological techniques already available for operational programs on a large scale. Both techniques necessitate the use of aerial release of insects if they are to be used in country or continent wide programs. New control techniques based on genetically modified insects or symbionts will also require aerial release of live insects [4].

Examples of recent application of this technique include the combined release of parasitoids (*Diachasmimorpha longicaudata* mainly) and sterile males of *Ceratitis capitata* (1.5 billion pupae produced per week) within the Moscamed regional program (Mexico and Guatemala) [5]. This program eliminated medflies from more than 7.000 km^2^ including the state of Chiapas in Mexico [6], and is currently progressing into southern Guatemala.

Similarly, an integrated campaign combining insecticide treatment of cattle, insecticide targets and the release of sterile males eradicated tsetse (*Glossina austeni*) from Zanzibar [7], [8]. This campaign was based on the use of cardboard carton boxes. However, although paper bags (used for fruit flies) or carton boxes are made of biodegradable materials, they still constitute visual pollution. More importantly, they are expensive, require a lot of space in the aircraft, can collapse, and make it very difficult to control the environmental conditions to which the sterile males are exposed, thus reducing their quality [9]. Therefore, aerial release is more and more often achieved through releasing chilled adult sterile insects thanks to automatic aerial release machines on board small single or twin engine aircraft in most current SIT programs. Besides being the fastest method of release, it has been demonstrated to provide uniform distribution over the target areas and to help ensure sterile insect quality and survivability [9]. Aerial release machines are designed with four basic components, a cooling unit, a controler for the release rate, a release mechanism and a navigation system. Early release mechanisms used screw augers [10] which caused mechanical injuries drastically decreasing the survival of insects [11]. This was replaced later by a conveyor belt [12] to adjust and calibrate the rate and desired density by manual and mechanical devices handled by the pilot, and thereafter using automatic navigation systems [9]. The Moscamed program presently uses machines based on conveyor belts and designed by the Mubarqui Company. However, existing machines are not adapted to tsetse release because they can not achieve the very low release rates required (between 10 and 100 per km^2^ only).

In this paper, we describe the Mubarqui Smart Release Machine (MSRM), a new design for release machines by the Mubarqui Company, based on the use of vibrating conveyors that avoid damaging insects and allow a very large range of release densities. Moreover, the smaller model is only 64 kg and can be accommodated on board gyrocopters (table 1), which will reduce the costs of aerial release, particularly in small target areas [13]. It is presently in use in the tsetse eradication campaign in Senegal (http://www.fao.org/news/story/en/item/211898/icode/). The machine is calibrated by means of a Geographic Information System (GIS) and environmental parameters are controlled using air conditioning.

**Table 1:** Types of release machines available as a function of the aircraft and species to be released.

Model	MSRM1	MSRM1	MSRM1	MSRM1	MSRM2
Type of aircraft	CESSNA 401 & 402	CESSNA 206 &207	Maule M7	CESSNA 172	GYROCOPTER
Species	*C. capitata* (7 to 9 mg fly)	*A. ludens* *A. oblique*(19 or 20 mg fly)	*A. ludens*	*C. capitata*	*G. palpalis gambiensis* (21 mg fly)
Capacity	60 million (3 machines)	6 million (1 machine)	6 million (1 machine)	5 million (1 machine)	15,000 (1 machine)
Minimum continuous release rate	400 f/ha	600 f/ha	600 f/ha	600 f/ha	50 f/km^2^
Maximum release rate	6000 f/ha	6000 f/ha	6000 f/ha	6000 f/ha	–
Program objectives	600–6000 f/ha	1250–5000 f/ha	1500 f/ha	2000 f/fa	10–100 f/km^2^
Swath	500 m	100 and 500 m	100 m	500 m	500 m
Speed	230 km/h	150 km/h	140 km/h	140 km/h	110 km/h
Temperature	4°C	4°C	4°C	4°C	7–8°C
Power needed	90A/24V	60A/12V	60A/12V	60A/12V	40A/12V
Mass (empty)	70 kg * 3	70 kg	70 kg	45 kg	64 kg
Program	MOSCAMED Mexico	MOSCAFRUT Mexico	MOSCAFRUT Mexico	NERETVA Croatia	Tsetse eradication Senegal

In the present paper, we compared the MSRM to the conventional conveyor machine presently in use in the Moscamed and Moscafrut programs in Mexico regarding dispersal and recapture rates of two species of fruit flies. We also provide preliminary data on the tsetse release rates that are used in the eradication program in Senegal.

## Materials and Methods

### Description of the Mubarqui Smart Release Machine (MSRM)

**Figure 1:**
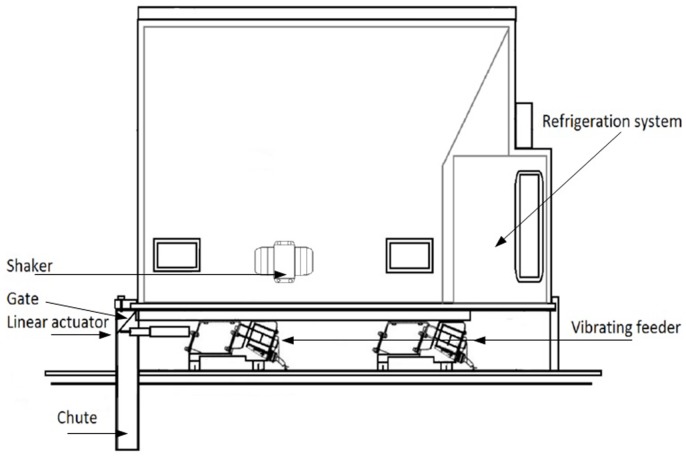
Plan of the version of Mubarqui Smart Release Machine (MSRM1) used to release fruit flies.

**Figure 2:**
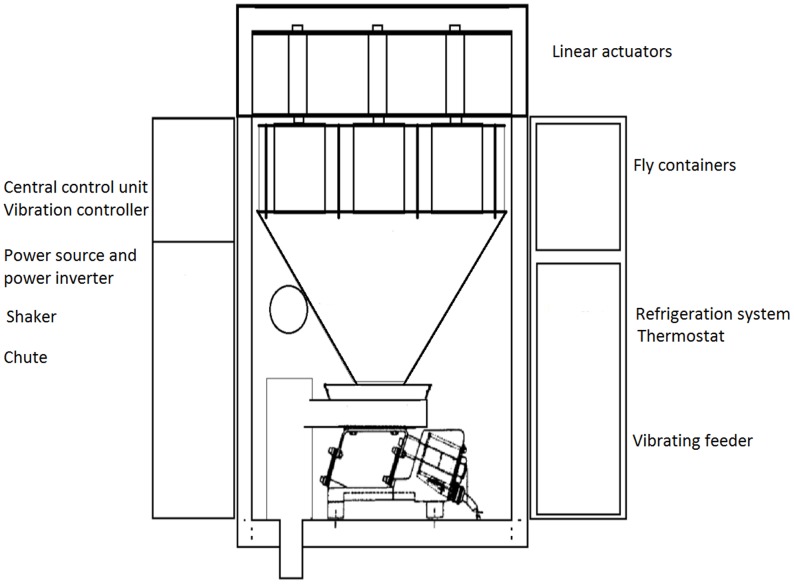
Plan of the version of Mubarqui Smart Release Machine (MSRM2) used to release tsetse flies.

The release device is installed inside an aircraft. It is designed with vibratory feeders, automatic gates and linear actuators with a duct leading the flies outside the aircraft, shaped to avoid Venturi suction in order to increase the accuracy of the release rate (see fig. 1 and fig. 2 for detailed plans of the machines used to release fruit flies (MSRM1) and tsetse flies respectively (MSRM2)). All the components are made of stainless steel. Unlike other machines, such as rotating conveyor or auger machines (previously used in Mexico), the smart machine has no longitudinal moving surface which is a great mechanical advantage that prevents physical damage to the insects. The material moves through micro vibration on a flat horizontal stainless steel tray that vibrates thanks to a powerful electromagnet moving horizontally 0.9 to 1.1 mm at 100 to 300 Hz. The release rate is controlled electronically by varying the frequency, power (acceleration) and displacement (amplitude) of this surface. These variables are operated by a digital controller and a program adapted to the different needs of the project (see Text S1), in this case the calibration of flies per hectare. The machine self-calibrates during the flight without the intervention of the pilot or other operator, thus avoiding human error. The gates are opened and closed automatically when entering or exiting the release area or when conditions are not appropriate for the material to be released.

The cooling system and container is a box with a refrigeration system that can be transported from the packing centers with the sterile adults to be released on a given day. This container is made of stainless steel and insulated from outside temperature to keep the biological material in suitable conditions for handling and transport both on the ground and in the air, to the polygon (area with a specific pre-defined release rate) where they will be released. It is equipped with a thermostat and vibrators to avoid air pockets within the mass of flies. Air pockets in the chilled flies must be avoided because it affects the regularity of the release rate [10].

The Central Control Unit receives instructions from the computer and turns them into actions such as opening and closing of the gates, start and stop release mechanism, intensity of the vibrating feeder, and modulation of micro vibrations to maintain accurate rates of release.

The Navigation system is the instrument responsible for guiding the pilot to the release areas (presented as polygons). It also contains the logging, measurement and automation for the release mechanisms.

Finally, the machine is equipped with video cameras connected to the computer, allowing the pilot to confirm that flies are being released.

A full description of operation and calibration of the MSRM is provided in Text S1. Figure S1 presents the installation of MSRM1 on board a Cessna 182 whereas Figure S2 presents its installation on board a Cessna 206 respectively. Figure S3 presents the installation of MSRM2 on board a gyrocopter in Senegal (aerodrome of Kalahari).

Two main types of machine are available according to the aircraft and species to be released (table 1). The tsetse machine is different from the two others in that it contains metallic cylindrical containers that are opened one by one to avoid any physical damage to the flies (fig. 2). The fruit fly machines can have containers of different sizes depending on the aircraft.

### Production and handling of the flies used in the trials

The two species of fruit flies (*Anastrepha ludens* and *Ceratitis capitata)* were mass reared at Metapa, Chiapas, Mexico [14], [15]. A genetic sexing strain producing males only was used for *C. capitata* [16], as well as for *A. ludens* (Tapachula 7) [17], reducing by 50% the cost of production, packing, and release.

Flies were packed, held and chilled in the Mexican type towers system [18], composed of 16 trays, each one of 81.77010.3 cm, including one container for pupae (55,000 *C. capitata* or 25,000 *A. ludens*), two trays for food (40 g for *C. capitata* and 20 g for *A. ludens*), one adult resting device and one pillow for water. For both species, mass rearing was achieved using the “Metapa system” [19]. Pupae were irradiated two days before emergence [20]. Pupae were then placed into a hopper and dispensed by means of a pupae dispenser machine which was then placed in trays specific to the tower and including all the other parts (feeding and resting devices). Flies were chilled at the sixth day after packing and then introduced into the release machines.

Regarding *Glossina palpalis gambiensis*, 74% of the pupae originated from Centre International de Rerche-Développement sur l’Elevage en zone Subhumide (CIRDES), Bobo-Dioulasso, Burkina Faso, and the remaining from the Institute of Zoology, Slovak Academy of Sciences, Bratislava, Slovakia. Females were bred in insectaries following standard procedures developed at CIRDES [21], [22]. They were kept at 24°C for female emergence and stored at 10°C when the first males emerged, leaving ∼96% of male pupae in this species. They were also transported in isotherm parcels containing phase change gel packs that maintain the temperature around 10°C. Pupae were permitted to emerge at the Institut Sénégalais de Recherche Agricole (ISRA) insectary in Dakar, maintained at 24°C and fed daily with cattle blood containing 10 mg/L of isomethamidium chloride (to avoid transmission of trypanosomes after release) [23]. They were chilled and placed into the MSRM four to six days after emergence.

### Field trials to compare MSRM to the conventional conveyor machine

A field trial was organized in 2012 in north-eastern Mexico (San Luis Potosi State, long. 21.937921°, lat. −100.097798) to compare the performance of the smart release machine with a vibrating feeder (MSRM1) to that of the release machine with a flat conveyor (MCRM). Two polygons of 600 ha (MSRM1) and 735 ha (MCRM) separated by 6 km from each other and comprised of citrus tree orchards were used for aerial releases of *A. ludens*. Densities of 2000, 1500, 1000 and 500 flies per ha were released each week for ten weeks, corresponding to a total duration of 39 weeks (only 9 weeks for the last density) in 2012. The swath width was 150 m. Twenty-four and 30 protein baited traps (McPhail) were set in the first and second polygons (corresponding to 0.04 trap per ha) and emptied weekly.

Another trial was organized in Chiapas State (long. 15.263055°, lat. −92.778553), Mexico, using *C. capitata*. The same protocol was used except that the polygons were 3000 ha each and separated by 37 km. The same densities were used but the trial was 20 weeks only (5 weeks for each density). The swath width was 500 m. Thirty-five and 33 traps were set respectively in the MSRM1 and MCRM polygons (corresponding to ∼0.01 trap per ha).

The number of flies packed into the device was different between release densities, to mimic a real situation within an eradication project, where the machine load depends on the objective in terms of release densities.

Finally, a third trial was organized in the Niayes area of Senegal (long. −17.1294, lat. 14.7831), using *G. p. gambiensis*, from Dec. 2013 to May 2014. Releases using paper boxes were compared to MSRM2. At the beginning of the trial (7 weeks), paper boxes were kept at ambient temperature (measured with a Hobo thermo-hygrometer station at 13–31°C) and then (8 weeks) within ice-boxes containing phase change gel packs to control the temperature (14–25°C). In MSRM2, we set the temperature inside the machine at 6–10°C for 6 weeks and at 9–12°C for 5 weeks. All the trials were conducted in a polygon of 8100 ha and we used 19 biconical traps to monitor tsetse densities [24]. The swath width was 500 m. Paper boxes were released every 500 m.

All the raw data from these trials are available in Suporting data S1, both at the trap and polygon level.

All trial areas are under SIT control (aerial releases), for more than five years in Mexico, and for two years in Senegal. In San Luis Potosi and Chiapas states, federal government through SENASICA Moscafrut and Moscamed respectively as agriculture authorities and DGAC as aeronautic authorities gave necessary permissions to fly and to release sterile males to implement these control programs. In Senegal, the trials were supervised by the Vet services within the tsetse eradication campaign which received the permit n°0874/MEPN/DE/DEIE/mbf from the Ministry of Environment on 03 April 2012. No specific permissions were required for the research activities presented in this paper, which were implemented within the control areas. The field studies did not involve endangered or protected species.

### Statistical methods

R software [25] was used for all statistical analyses.

The rate of positive traps (capturing at least one fly) were analyzed using generalized binomial models, with the release density, the type of release machine and their interaction as fixed effects.

The recapture rates of individual traps were analyzed using generalized linear mixed binomial models [26] using the release density, the type of release machine and their interaction as fixed effects and the trap position as a random effect [27]. In addition, in the case of *C. capitata*, the tree species on which the traps were set (which were all citrus tree in the *A. ludens* trial) was a fixed effect. In the case of tsetse, the release method (paper boxes vs MSRM2 and temperature regimen) and the release density were considered as fixed effects. The lme4 package was used to implement these models [28].

The best model was selected based on the lowest corrected Akaike information criterion (AICc), [29] using the MuMin package [30].

## Results

### Anastrapha ludens

**Figure 3:**
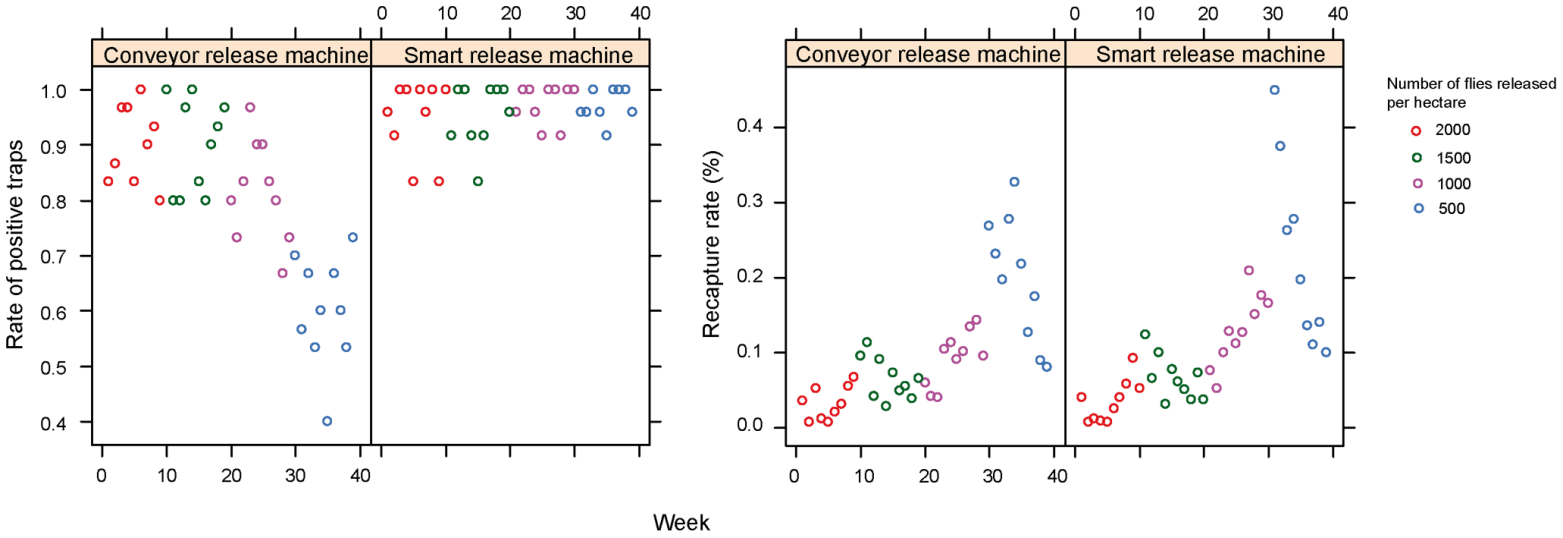
Comparison of conveyor (MCRM) and vibrator (MSRM1) release machines for *Anastrepha ludens* in Mexico. Rate of positive traps (left) and recapture rates (right) are presented for different release densities. A trap is considered positive when at least one fly is captured during a week.

There was a strong positive impact (p<0.001) of release density on the percentage of positive traps (fig. 3 and Table S1). Moreover, the rate of positive traps was higher for MSRM, and the difference was higher at low release densities (1000 per ha and lower, p<0.001).

There was also a strong negative impact (p<0.001) of release density on the percentage of recaptured flies (fig. 3 and Table S1). Moreover, the percentage of recapture was higher for MSRM, and the difference was higher at low release densities (p<0.001).

### Ceratitis capitata

**Figure 4:**
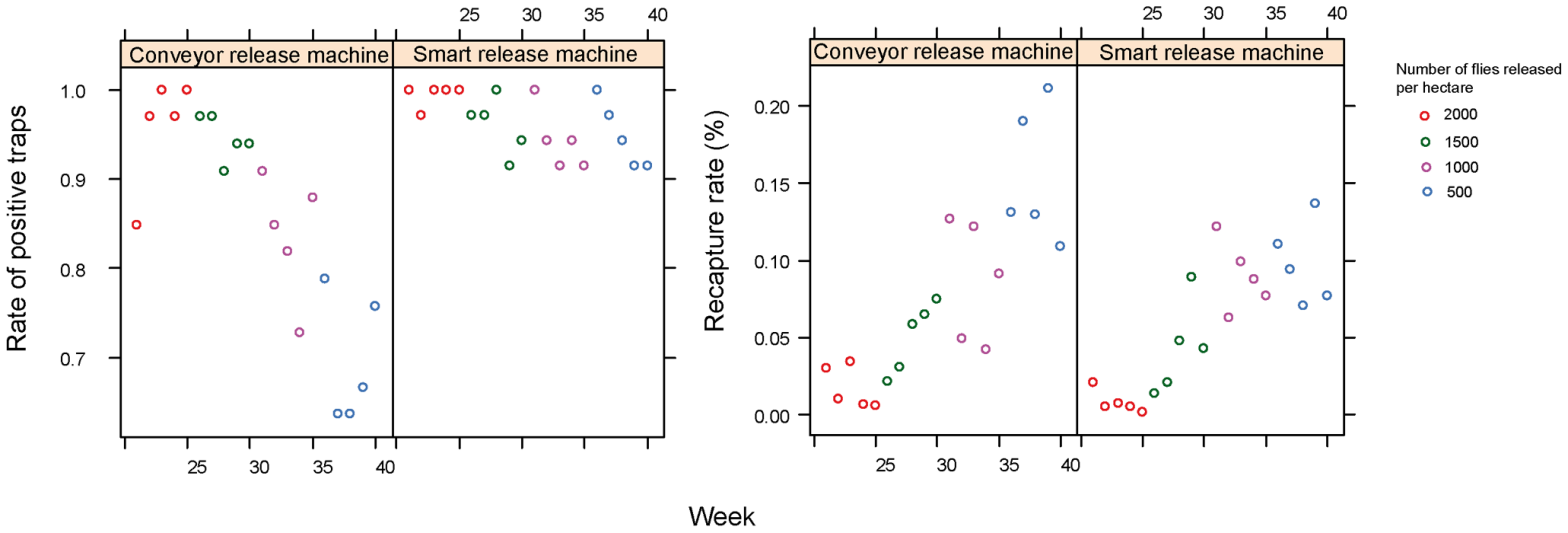
Comparison of conveyor (MCRM) and vibrator (MSRM1) release machines for *Ceratitis capitata* in Mexico. Rate of positive traps (left) and recapture rates (right) are presented for different release densities. A trap is considered positive when at least one fly is captured during a week.

For *C. capitata* as well as for *A. ludens*, there was a strong positive impact (p<0.001) of release density on the percentage of positive traps (fig. 4 and Table S2). Moreover, the rate of positive traps was higher for MSRM, but the difference was significant only at low release densities (1000 per ha and lower, p<0.001).

There was a strong negative impact (p<0.001) of release density on the percentage of recaptured flies (fig. 4 and Table S2). There was no significant difference between the two release machines on the recapture rate (p = 0.72). However, this rate of recapture decreased more with density for the MSRM (p<0.001).

### Glossina palpalis gambiensis

There was a significant negative impact of release density on the recapture rate (p<0.05), but not on the percentage of positive traps (p = 0.14).

**Figure 5:**
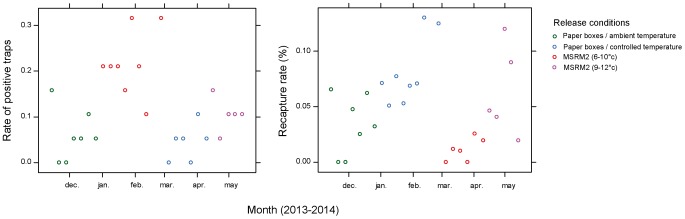
Comparison of paper boxes and vibrator release machine (MSRM2) for *Glossina palpalis gambiensis* in Senegal. Rate of positive traps (left) and recapture rates (right) are presented for different release conditions: paper boxes at ambient temperature (13–31°C), paper boxes at controlled temperature (14–25°C), MSRM2 with the temperature controlled at 6–10°C or 9–12°C. A trap is considered positive when at least one fly is captured during a week.

The percentage of positive traps was similar (p>0.14) between release methods (fig. 5 and Table S3) except that it was lower (p = 0.02) for MSRM2 used with a temperature range of 6–10°C.

The recapture rate was better for the paper boxes in controlled temperature conditions than all other methods (p<0.05). There was no difference between paper boxes maintained at ambient temperature and the MSRM2 used with a temperature range of 6–10°C (p = 0.34). However, MSRM2 9–12°C gave better results than paper boxes at ambient temperature (p = 0.05) and MSRM2 6–10°C (p = 0.005).

### Possible tsetse release rates for MSRM2

**Figure 6:**
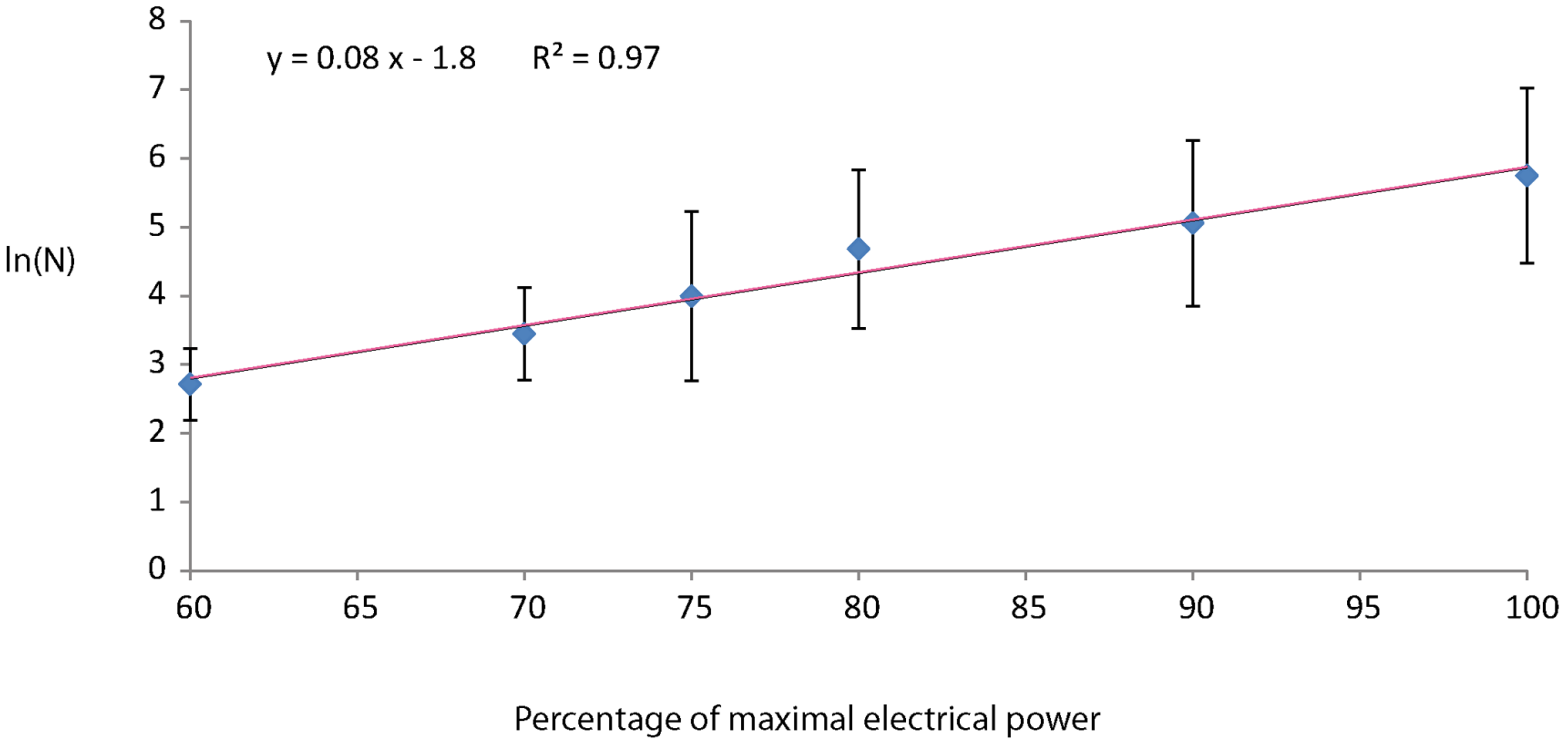
Parameterization of release rates of *Glossina palpalis gambiensis* with the vibrator release machine (MSRM2). The graph presents the linear correlation between the natural logarithm of the number released by second and the power of the vibrator (as a percentage of the maximal electrical power). Vertical bares present the standard errors (three repeats of 1 minute per value). This test was achieved within the tsetse eradication program in Senegal (http://www.fao.org/news/story/en/item/211898/icode/).

In Senegal, the total target area is only about 1000 km^2^ [31]. There was a very good correlation (p<10^–3^) between the natural logarithm of the number of tsetse flies released by second and the power of the vibrator (fig. 6). The minimum adjustment rate obtained with MSRM2 was ∼50 flies/km^2^ (200 times lower than for fruit flies) with continuous release but the tsetse eradication program requires even less, down to 10 flies/km^2^ (0.1 fly per ha). This was obtained by alternating periods of turning on and off the vibrator (the turning off cannot exceed 18 seconds because the distance between releases must remain lower than the swath width to ensure good dispersal, which is 500 m in this project, whereas the release speed of the gyrocopter is 110 km/h).

**Figure 7:**
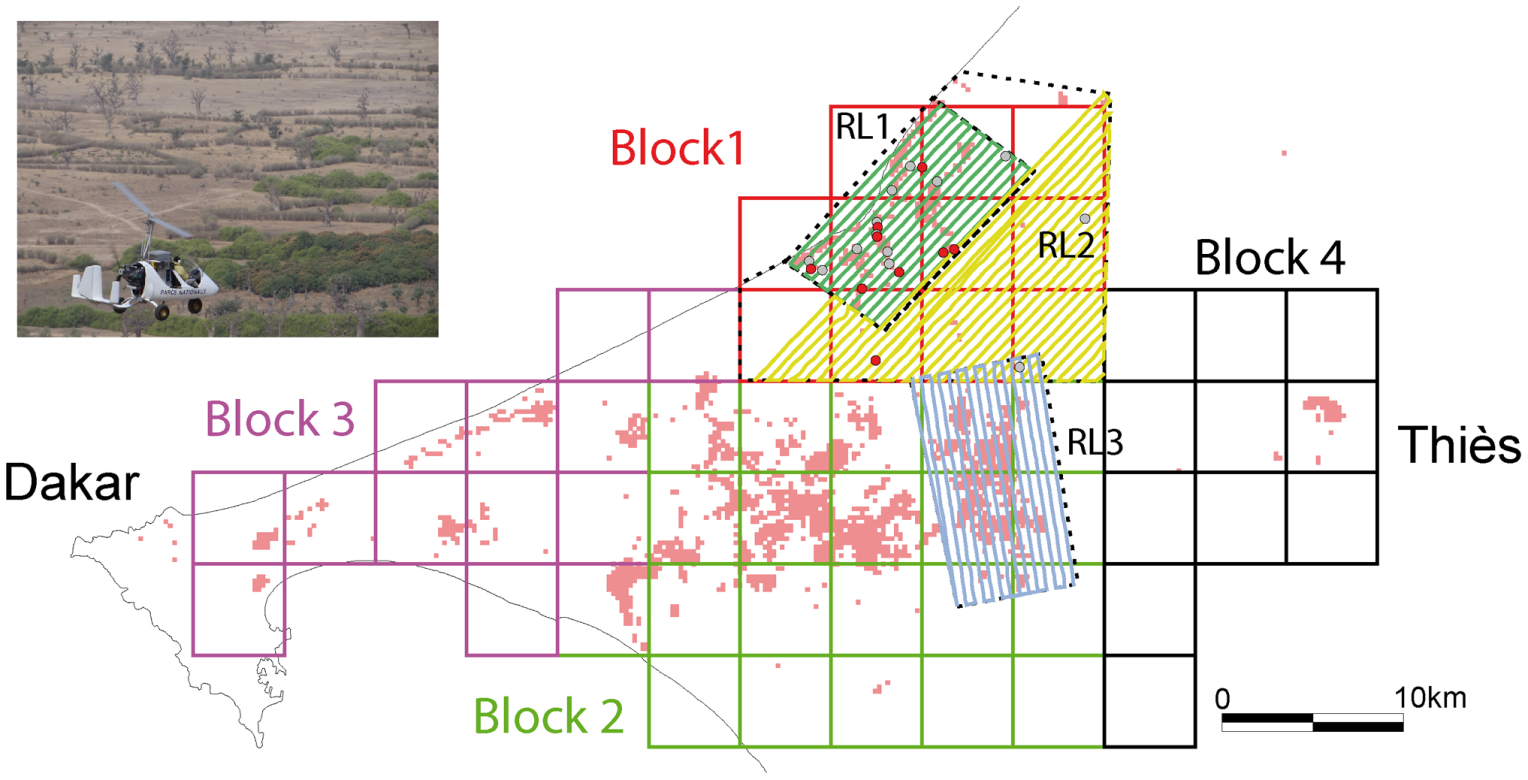
Release patterns of chilled male *Glossina palpalis gambiensis* in Senegal. The control area is subdivided in four blocks targeted sequentially and each block is subdivided in polygons where the release density is adjusted according to the amount of suitable habitats. Since 28 February 2014, all releases of sterile flies are achieved using MSRM2 on board a gyrocopter of the Kalahari aerodrome (picture at the top left of the figure). The minimal release rates are 0.26 flies per ha for RL1, 0.12 flies per ha for RL2 and 0.53 flies per ha for RL3 and actual release rates are adjusted to the availability of flies at the release center (ISRA insectarium, Dakar-Hann, Senegal). The tracks presented on the figures correspond to the flights of 21 March 2014 for RL1, 05 April 2014 for RL2 and 15 April 2014 for RL3. Grey points represent trap positions where no tsetse was captured in April-May 2014 whereas red points present trap positions where sterile males released with MSRM2 were recaptured.

Since 28 February 2014, all releases of sterile flies in the Senegal tsetse eradication program have been achieved using MSRM (fig. 7). Overall, 126, 975 flies have been released by the time of writing. The minimal release rates are 0.26, 0.12 and 0.53 flies per ha for RL1, RL2 and RL3 respectively. Release rates are, however, adjusted on a daily basis according to the availability of flies. RL1 is the polygon where the comparison experiments described upon were conducted (fig. 7). Mean number of flies released per week is 14,346 (s.d. 2,397). A video camera allowed confirmation of a very regular release rate of the flies.

### Discussion and perspectives

The first objective of this study was to compare a new release system for chilled adult release, the MSRM using a vibratory feeder instead of a conveyor belt, presently used in MCRM within the Moscamed and Moscafrut programmes in Mexico. The MCRM, provides good results at high release densities for fruit flies [10], but it is relatively inaccurate at low densities. The second objective was to evaluate the possibility of releasing tsetse flies with the same system because no release machine had previously been available for tsetse. MCRM2 appeared to offer an effective means to automatically and aerially release tsetse and we thus compared its efficiency to the standard method (paper boxes at ambient temperature [7]) and an improved method (paper boxes at controlled temperature).

The comparison of MSRM1 and MCRM gave similar results for the two fruit fly species tested. The rate of positive traps gives a good picture of fly distribution due to aerial release [9]. The results show that the MSRM performs better than the conveyor release machine for both fruit fly species, especially at low density, showing a more regular release rate for this machine. Since the same guidance system (MaxNav) was used for both machines during these trials, the better results of MSRM1 can be attributed to its mechanical properties only. This parameter is clearly the most important in eradication programs, since the sterile to wild male ratios must be as homogeneous as possible to induce female sterility over the entire target area [9]. At a release rate of 1500 flies per ha, the two machines also gave much better results (∼95% of positive traps) than the auger release systems (70 and 80% of positive traps for the two models tested in [9]).

Tween & Pendon (2007) suggest that the recapture rate is related to longevity or survival and attribute this to fly handling during release activities [9]. Other parameters (trapping intensity and fly release density) were the same in the two release polygons. Mortality (here considered as both true mortality and emigration from the target area), was highly density dependent in this trial. This increased mortality (lower recapture rate) at higher release densities could result from cramped storage in the release device due to the large number of flies used (up to 1.2 million *A. ludens* and 6 million *C. capitata*, corresponding to ∼22 kg and ∼48 kg respectively). Moreover, the recapture rate was higher for the MSRM for *A. ludens*, especially at low densities (up to 38% higher for 500 flies per ha). For *C. capitata*, however, no significant difference was observed between the two machines. The two machines had similar efficiencies at high densities (more than 1500 per ha).The mean survival for *A. ludens* is ∼15 days, causing an accumulation effect which might have created a bias for the first two weeks after a change in release densities. This however did not prevent us from observing a strong signal between series.

In the MOSCAMED project in Mexico, the minimum density of flies to be released is 500 fly/ha (50,000/km^2^), whereas MSRM1 could be adjusted down to 100 flies/ha (10,000/km^2^) with continuous release for *C. capitata* (table 1). For fruit flies, the intensity of the vibrator has to be adjusted to the stage of the flies available at each daily collect (humid, semi humid or dry flies), because the behavior is different regarding release rates. In the case of tsetse, recapture rates and percentages of positive traps were lower than for fruit flies for all release methods. This was expected because tsetse traps are based on visual stimuli and capture rates are always very low [32]. In the target area, trap efficiency was estimated to be ∼0.003 per day per km^2^ [33]. MSRM2 with a temperature range of 9–12°C outperformed standard release procedures using paper boxes at ambient temperature (13–31°C), which was used to eradicate *G. austeni* from Zanzibar [7]. However, the recapture rate of MSRM2 with a temperature range of 9–12°C was still slightly lower than with paper boxes in controlled temperature conditions (14–24°C). One possible explanation for this result is that some flies do not become active before reaching the ground using MSRM2. We estimated the maximal speed of cold live flies at 3.2 m/sec (s.d. 0.1) by filming their free fall in front of a white wall. Based on this, it is likely that released tsetse reach the ground within 31 seconds from the release altitude of 100 m in Senegal. However, we also observed using a camera that flies kept at 10°C take 30 seconds to 1 min to wake up at 30°C. Some of them might thus hit the ground and be predated before being able to fly. We will make release trials at higher altitudes (200 m) in the future to try to improve the recapture rate. An alternative hypothesis might be that low temperatures decrease adult tsetse survival, which might explain the better results of the 9–12°C range than the 6–10°C range for MSRM2 (although flies also wake up faster at higher temperatures). It is however impossible to increase temperature furthermore within MSRM2 because upon 12°C, we observed that flies can attach to the stainless steel components thanks to their legs which affects control of release rates.

MSRM2 offers the first opportunity to use the chilled insect release technique in tsetse, where the low release rates needed and the high sensitivity of the flies made it impossible to use auger machines. The use of a gyrocopter reduces flying costs to €320/hour. The speed used with this machine for aerial release was 110 km/h. MSRM2 fulfilled the requirement of the tsetse eradication program in Senegal (http://www.fao.org/news/story/en/item/211898/icode/) for very low release rates (0.1–0.5 flies per ha presently) and has been used routinely since 28 February 2014 to replace in replacement of the boxe. This is also because only ∼100 paper boxes can be carried on board a gyrocopter, which limits the release area that can be covered within one flight with this release method. During the field trials in Senegal, no dead flies were observed in the machine after release and the number of flies remaining in the machine was below 50. In the case of tsetse flies, it was demonstrated that when released in a homogeneous density, sterile males are able to aggregate in the same sites preferred by wild flies [34].

The MSRM maintains stable environmental conditions inside the machine thanks to the absence of suction, which limits loss of chilled air. The main drawback is that the machine necessitates a permanent source of power, which can be challenging for small aircraft such as gyrocopters. In single engine aircrafts, the main power source is enough (12V, 100A). In twin engine, the power source is even stronger (24V, 150A). In Senegal however, the use of a gyrocopter was more challenging and we used an auxiliary power unit (APU, 12V, 65A) and an intermediate battery to provide enough power to the cooling system.

We plan to test the machine on parasitoids (*Diachasmimorpha* species), which are more sensitive than other insect species particularly because of long antennae (Montoya et al., 2012). MSRM will also be tested on mosquito species (*Aedes albopictus* in particular) for which the sterile insect technique is still at the R&D stage [35], [36], and for which a new concept called “boosted SIT” has recently been developed, where sterile males are used as specific conveyors of active biocides [37]. Since mosquitoes are very sensitive to mechanical injury [38], the vibrating feeder might provide a technical solution for their release, but this hypothesis remains to be tested.

## Supporting Information

Figure S1 **Installation of MSRM1 on board a Cessna 182.**
(TIF)

Figure S2 **Installation of MSRM1 on board a Cessna 206.**
(TIF)

Figure S3 **Installation of MSRM2 on board a gyrocopter in Senegal (aerodrome of Kalahari).**
(TIF)

Table S1 **Recapture rate and rate of positive traps for different release densities of** ***Anastrepha ludens*** **in Mexico.** Standard deviations are presented in brackets. Each value was estimated from 10 measures for all release densities except 500 (9 measures).
(DOCX)

Table S2 **Recapture rate and rate of positive traps for different release densities of** ***Ceratitis capitata*** **in Mexico.** Standard deviations are presented in brackets. Each value was estimated from 5 measures.
(DOCX)

Table S3 **Recapture rate and rate of positive traps for** ***Glossina palpalis gambiensis*** **in Senegal.** Standard deviations are presented in brackets.
(DOCX)

Text S1 **Calibration and operating of the Mubarqui Smart Release Machine.**
(DOCX)

Data S1 **Data of release-recapture trials in Mexico (Excel 2010 file).**
(XLSX)
