# Supplementary figures and images for: The Smart Aerial Release Machine, a Universal System for Applying the Sterile Insect Technique
Source: PLoS One. 2014 Jul 18;9(7):e103077. doi: 10.1371/journal.pone.0103077 (PMC4103892; doi:10.1371/journal.pone.0103077)

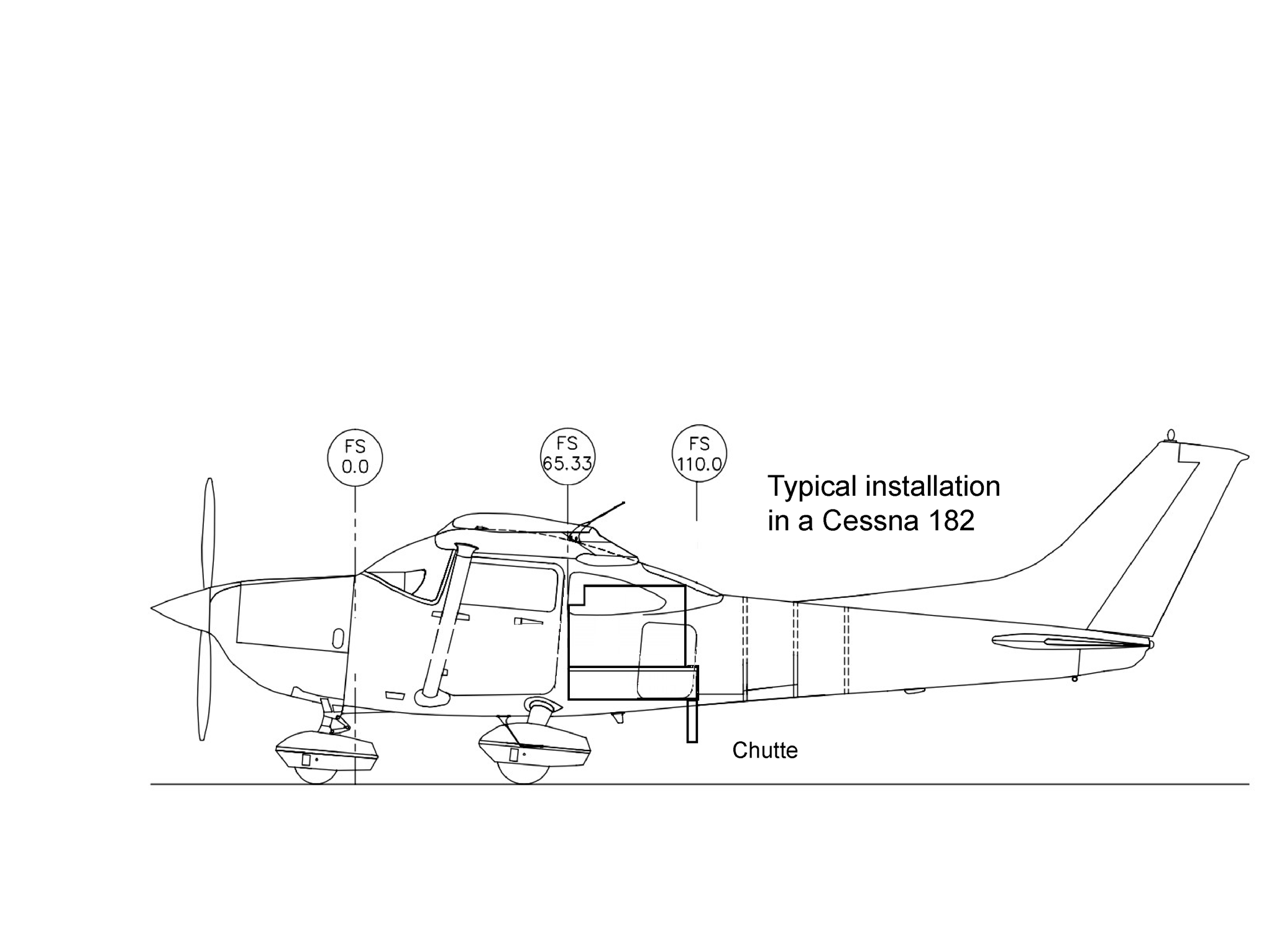

Supplement: Figure S1 — Installation of MSRM1 on board a Cessna 182. (TIF) [file pone.0103077.s001.tif]

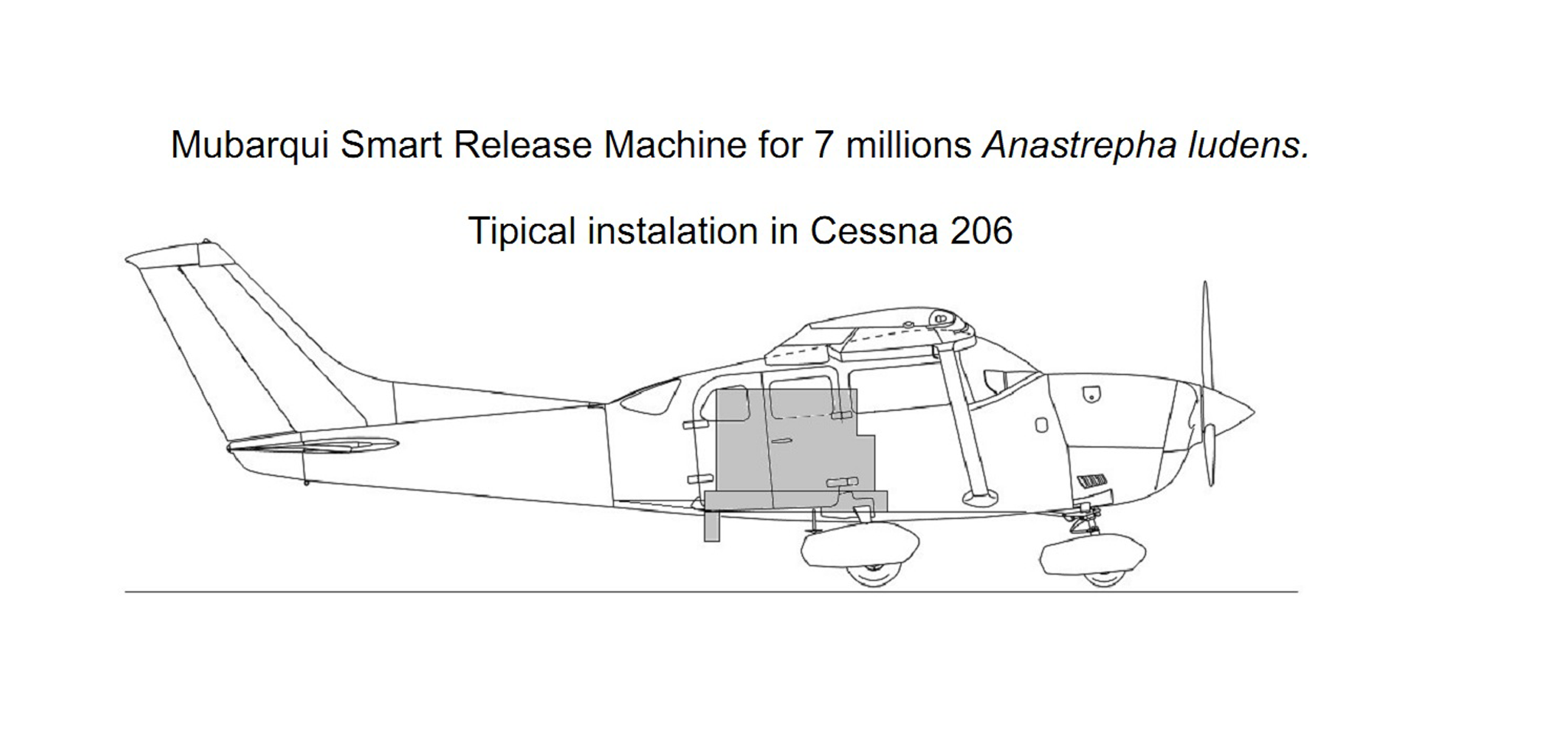

Supplement: Figure S2 — Installation of MSRM1 on board a Cessna 206. (TIF) [file pone.0103077.s002.tif]

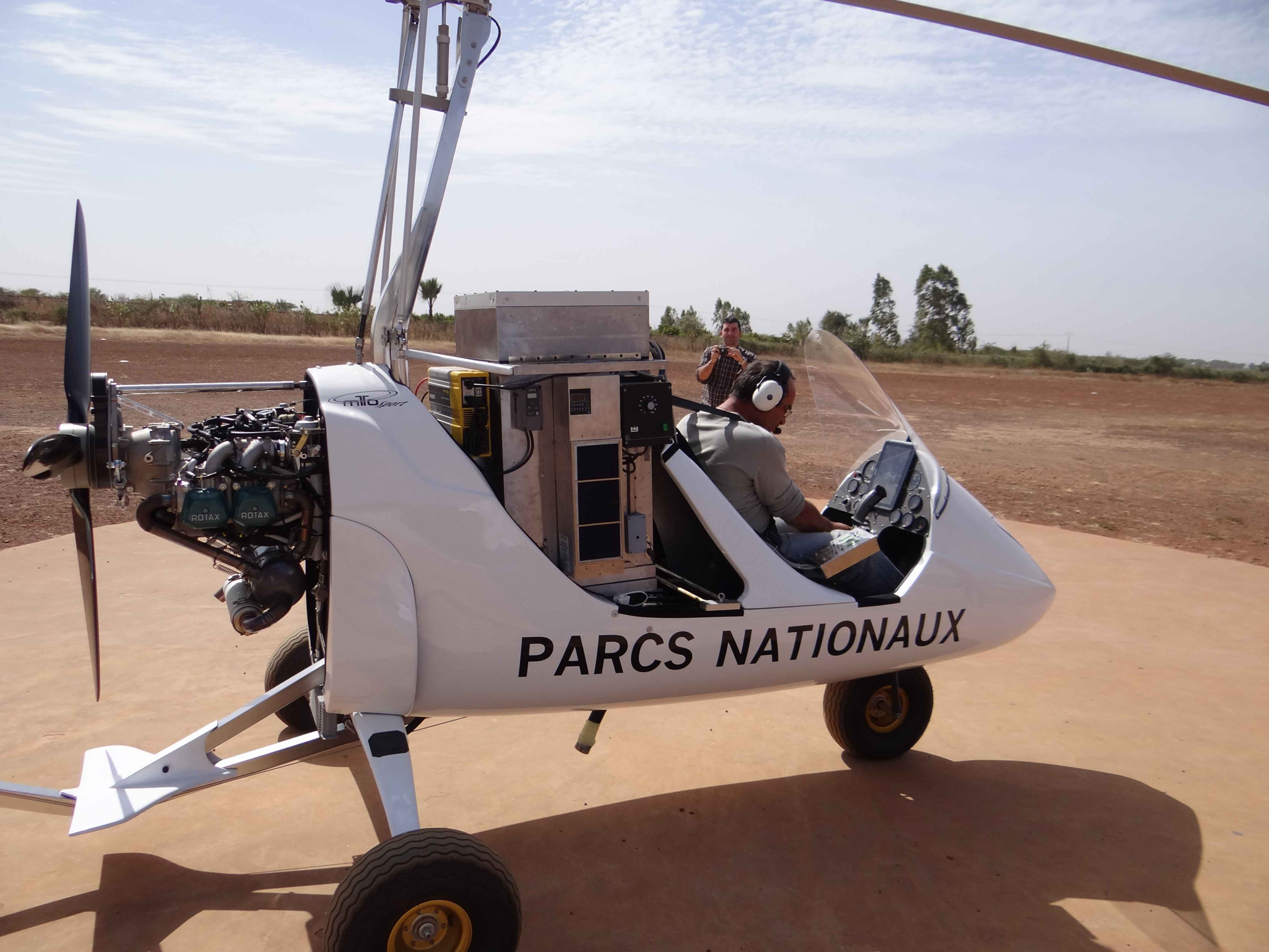

Supplement: Figure S3 — Installation of MSRM2 on board a gyrocopter in Senegal (aerodrome of Kalahari). (TIF) [file pone.0103077.s003.tif]
