# Supplementary material for: The Smart Aerial Release Machine, a Universal System for Applying the Sterile Insect Technique
Source: PLoS One. 2014 Jul 18;9(7):e103077. doi: 10.1371/journal.pone.0103077 (PMC4103892; doi:10.1371/journal.pone.0103077)
Supplement: Table S1 — Recapture rate and rate of positive traps for different release densities of Anastrepha ludens in Mexico. Standard deviations are presented in brackets. Each value was estimated from 10 measures for all release densities except 500 (9 measures). (DOCX) [file pone.0103077.s004.docx]

|  | Recapture rate (%) | | Rate of positive traps | |
| --- | --- | --- | --- | --- |
| Release density (flies per ha) | MCRM | MSRM1 | MCRM | MSRM1 |
| 500 | 0.20 (s.d. 0.08) | 0.23 (s.d. 0.12) | 0.60 (0.10) | 0.97 (0.03) |
| 1000 | 0.09 (s.d. 0.04) | 0.13 (s.d. 0.05) | 0.82 (0.10) | 0.98 (0.04) |
| 1500 | 0.07 (s.d. 0.03) | 0.07 (s.d. 0.03) | 0.90 (0.08) | 0.95 (0.06) |
| 2000 | 0.03 (s.d. 0.02) | 0.03 (s.d. 0.03) | 0.90 (0.07) | 0.95 (0.07) |
