# Supplementary material for: The Smart Aerial Release Machine, a Universal System for Applying the Sterile Insect Technique
Source: PLoS One. 2014 Jul 18;9(7):e103077. doi: 10.1371/journal.pone.0103077 (PMC4103892; doi:10.1371/journal.pone.0103077)
Supplement: Table S2 — Recapture rate and rate of positive traps for different release densities of Ceratitis capitata in Mexico. Standard deviations are presented in brackets. Each value was estimated from 5 measures. (DOCX) [file pone.0103077.s005.docx]

|  | Recapture rate (%) | | Rate of positive traps | |
| --- | --- | --- | --- | --- |
| Release density (flies per ha) | MCRM | MSRM1 | MCRM | MSRM1 |
| 500 | 0.15 (s.d. 0.04) | 0.10 (s.d. 0.03) | 0.70 (s.d. 0.07) | 0.95 (s.d. 0.04) |
| 1000 | 0.09 (s.d. 0.04) | 0.09 (s.d. 0.02) | 0.84 (s.d. 0.07) | 0.94 (s.d. 0.03) |
| 1500 | 0.05 (s.d. 0.02) | 0.04 (s.d. 0.03) | 0.95 (s.d. 0.03) | 0.96 (s.d. 0.03) |
| 2000 | 0.02 (s.d. 0.01) | 0.01 (s.d. 0.01) | 0.96 (s.d. 0.06) | 0.99 (s.d. 0.01) |
