# Supplementary material for: The Smart Aerial Release Machine, a Universal System for Applying the Sterile Insect Technique
Source: PLoS One. 2014 Jul 18;9(7):e103077. doi: 10.1371/journal.pone.0103077 (PMC4103892; doi:10.1371/journal.pone.0103077)
Supplement: Table S3 — Recapture rate and rate of positive traps for Glossina palpalis gambiensis in Senegal. Standard deviations are presented in brackets. (DOCX) [file pone.0103077.s006.docx]

| Release density  (flies per ha) | Release device | Temperature range (°C) | Recapture rate (%) | Rate of positive traps | Number of repeats |
| --- | --- | --- | --- | --- | --- |
| 0.46 (s.d. 0.18) | Paper boxes | 13-31 | 0.03 (s.d. 0.03) | 0.06 (s.d. 0.06) | 7 |
| 1.06 (s.d. 0.17) | Paper boxes | 14-25 | 0.08 (s.d. 0.03) | 0.22 (s.d. 0.07) | 8 |
| 0.85 (s.d. 0.34) | MSRM2 | 6-10 | 0.01 (s.d. 0.01) | 0.04 (s.d. 0.04) | 6 |
| 0.80 (s.d. 0.28) | MSRM2 | 9-12 | 0.06 (s.d. 0.04) | 0.11 (s.d. 0.04) | 5 |
