# Supplementary material for: The Smart Aerial Release Machine, a Universal System for Applying the Sterile Insect Technique
Source: PLoS One. 2014 Jul 18;9(7):e103077. doi: 10.1371/journal.pone.0103077 (PMC4103892; doi:10.1371/journal.pone.0103077)
Supplement: Text S1 — Calibration and operating of the Mubarqui Smart Release Machine. (DOCX) [file pone.0103077.s007.docx]

**Supporting Information 1: Calibration and operating of the Mubarqui Smart Release Machine**

***Before the flight***

Release parameters, i.e. altitude, swath width (flight line spacing) and density must be defined by the technical staff and stored in the online MACX SYSTEM database. Interactive pages allow scheduling flights by issuing a release order.

The following parameters have to be specified for each polygon of the target area in the database:

- Polygon name,
- Length, width and area of the polygon (in hectares),
- Position and geographic boundaries (specified as a GIS layer),
- Elevation,
- Separation lines and release lines (specified as a GIS layer),
- Release density ,
- Maximum and minimum altitudes for release.

These specifications are needed to calculate the release rate when entering into a release polygon depending on position, speed and latitude. The release rate is defined by the formula MAVZ, where M = adults per ha, A = swath width in meters, V = ground speed in km/h and Z = 0.0000278 (correction factor).

The establishment of the release order based on the polygon properties requires the following additional data:

- Name of target polygons for a given flight,
- Number of flies collected for flight,
- Release flight time, ferry time and total flight time.

The release order for the MSRM can be transmitted to the tablet via internet since the same format is used in the database and flight computer. These data are downloaded just before the flight, and give the navigation instructions to the pilot to fly, and to the Central Control Unit (fig. 1).


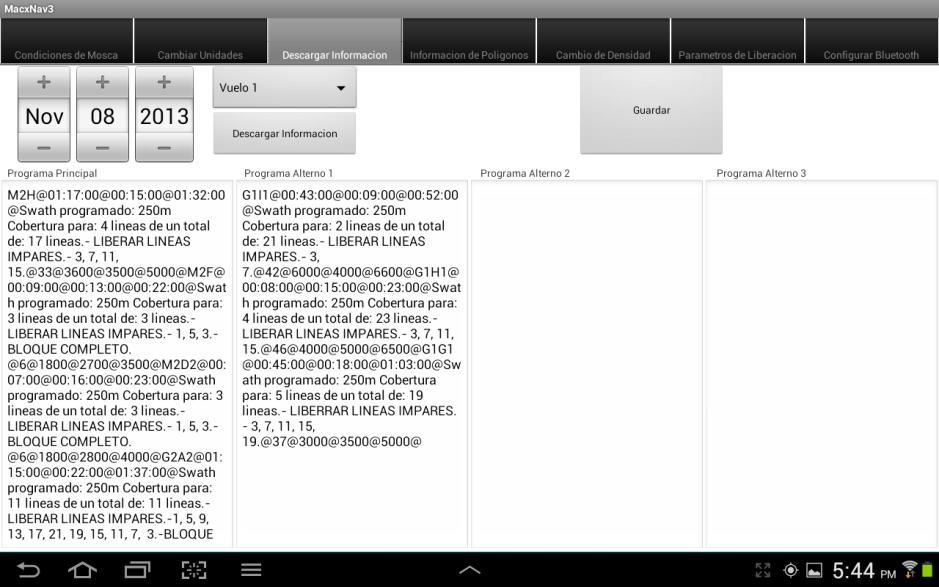


*Figure 1 Example of navigation instructions*

The Smart Release Machine uses software called MaxNav developed by the Mubarqui Company, which is installed in a tablet with the Android® Operating System, equipped with Global Positioning System (GPS). The access to this software is by request and agreement with the Mubarqui company. The tablet communicates via Bluetooth® with the Central Control Unit, and performs all actions necessary to release, such as starting releasing in target areas, stopping releasing in non-target areas, automatic calibration, increasing or decreasing the release rate for each polygon according to the plan. Exclusion or different density areas within polygons can be programmed through smaller polygons that will be automatically recognized by the device which can change the release rate in a dynamic way. This enables bigger polygons to be selected for the release lines, thus making savings on release time.

***Navigation***

On the tablet screen, the pilot has all the information needed to navigate, from the airport to the release area. The navigation instructions are provided by GPS technology through the flight computer, including speed, altitude, heading, track, flight times and separation of lines. Once the aircraft flies over the release polygon, the GIS compares speed, maximum and minimum release altitude and swath width of lines to the objectives planned and provides alarms for position, course, heading, altitude and speed if the pilot starts to deviate from the parameters indicated in the release order. If the indicators are outside the specified limits, it will stop the release. With the MSRM, the pilot thus has an additional navigator for the flight (fig. 2), adding greater security by allowing the pilot to focus on the flying operations and not give so much attention to the operation of the old style release machine for which, besides flying, the pilot has to be aware of the calibration, opening and closing the doors, turning on and off the device, recording the time of entry and exit of each polygon and finding the release line.


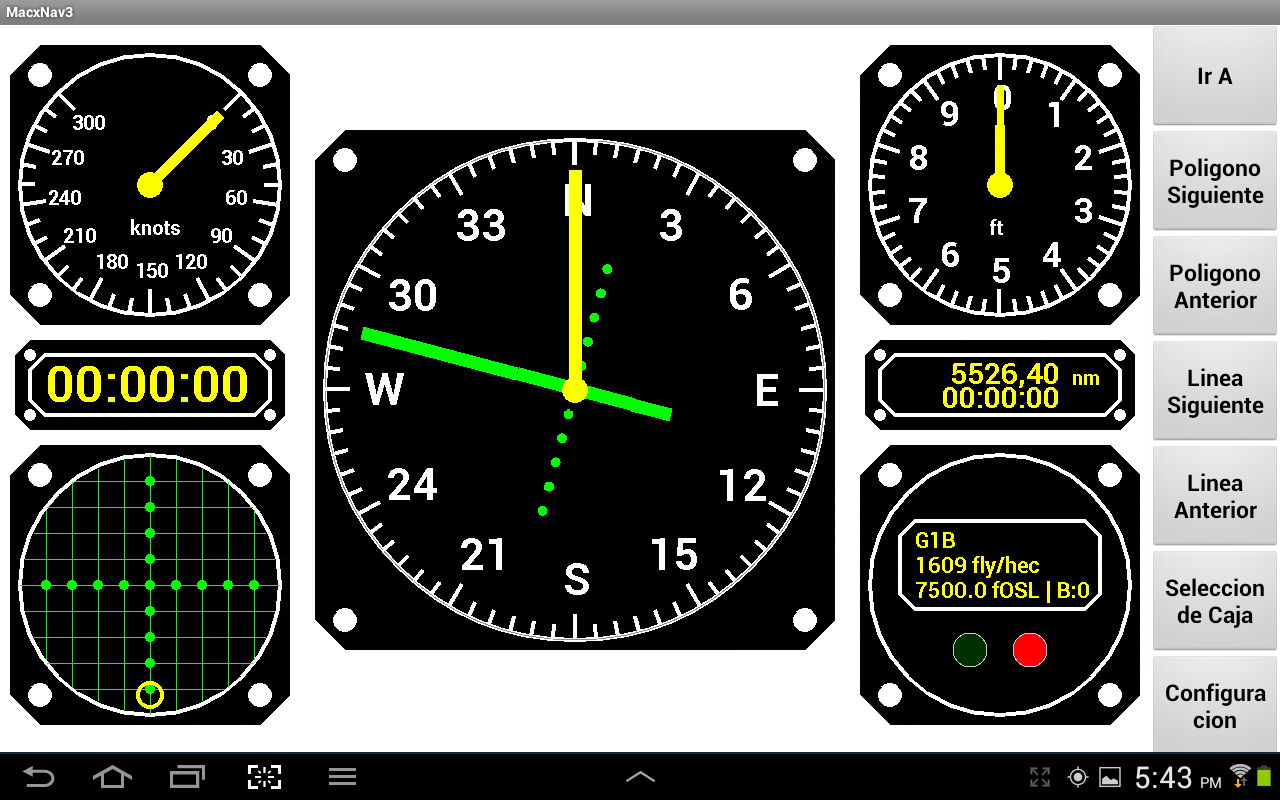


*Figure 2 Flight navigator available within MaxNav software*

When the tablet receives the release program of the day, potential alternate polygons are indicated in addition to the main release flight order, specifying the sequence in which the polygons should ideally be treated. Once the pilot is in effective command, he receives navigation instructions sequentially to reach each of the release polygons (fig. 3). As soon as each polygon is reached, the GIS initiates and calibrates releases according to the current program. In case the principal release flight order is impossible due to weather conditions, the pilot changes to a secondary release flight order with alternate polygons (or different polygon order) in order to avoid wasting the sterile flies by releasing them in another site of the target area.


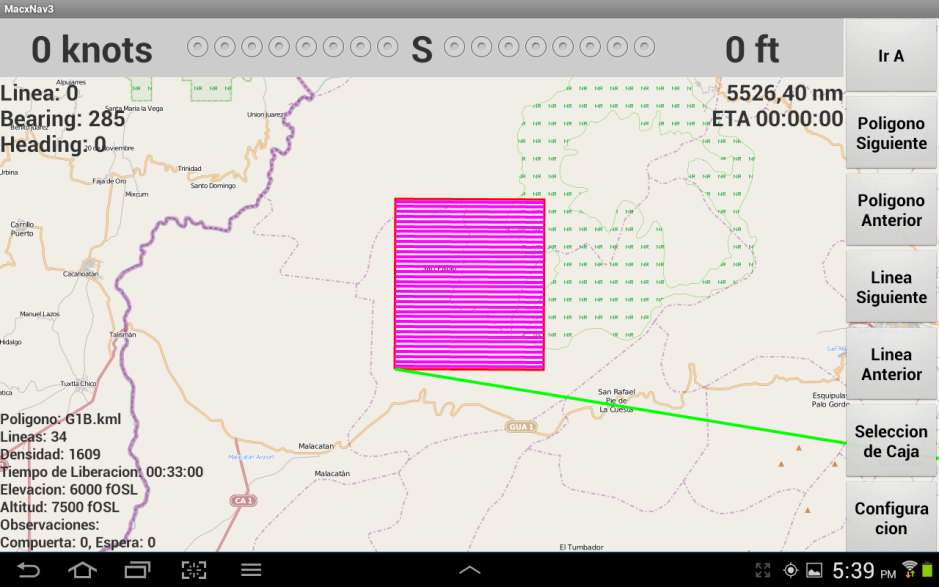


*Figure 3 Mapviewer included in the MaxNav system, guiding the pilot to the release polygon and then along the release lines in real time*

***Release report***

At the end of the release flight, the pilot synchronizes with the internet and uploads flying data in digital format to the MACX SYSTEM interactive website.

A release report is automatically generated by the program and includes:

• Date, time and duration of the flight,

• Polygons and hectares released,

• Name and registration number of the aircraft and pilot,

• Exact times for all events (start release, stop release, etc…),

• Path map of release (fig. 4),

• Altitude and average speed.


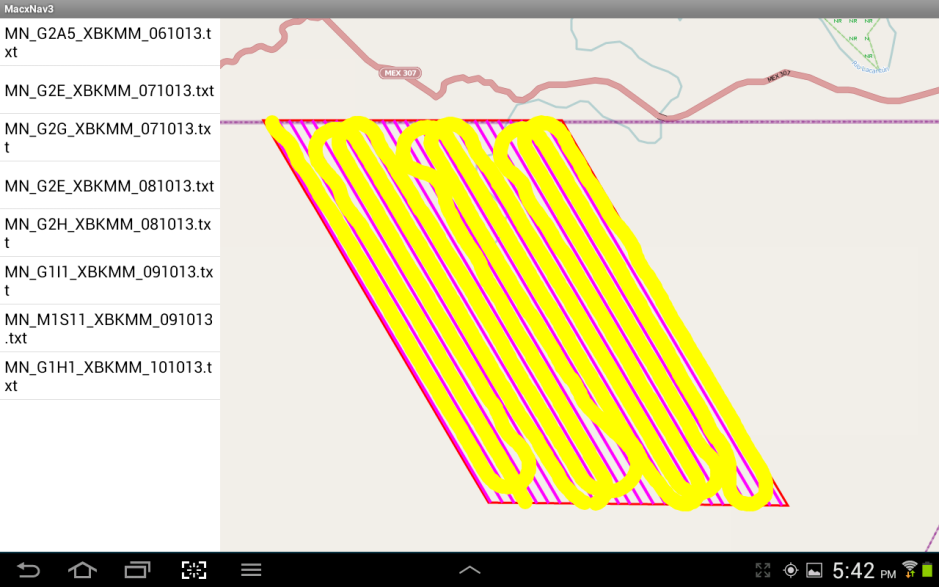


*Figure 4 Example of path map of release included in the automatic report.*

This report becomes available instantaneously by internet for review and statistical analysis by technical staff, field operators, programming teams, accountants and administrative staff without any transcription. The automatic report avoids human errors, saves time and paper, optimize resources and ensure the accuracy of the data. It can be added easily to the pilot flight report and delivered or sent to the right personnel. Most important, it maintains a digital database with all flights and allows comparison of the results with the capture results in the monitoring system and tracking and tracing of all actions taken.
